# Biofilm Formation by *Helicobacter pylori* and Its Involvement for Antibiotic Resistance

**DOI:** 10.1155/2015/914791

**Published:** 2015-05-19

**Authors:** Hideo Yonezawa, Takako Osaki, Shigeru Kamiya

**Affiliations:** Department of Infectious Diseases, Kyorin University School of Medicine, 6-20-2 Shinkawa, Mitaka, Tokyo 181-8611, Japan

## Abstract

Bacterial biofilms are communities of microorganisms attached to a surface. Biofilm formation is critical not only for environmental survival but also for successful infection. *Helicobacter pylori* is one of the most common causes of bacterial infection in humans. Some studies demonstrated that this microorganism has biofilm forming ability in the environment and on human gastric mucosa epithelium as well as on *in vitro* abiotic surfaces. In the environment, *H. pylori* could be embedded in drinking water biofilms through water distribution system in developed and developing countries so that the drinking water may serve as a reservoir for *H. pylori* infection. In the human stomach, *H. pylori* forms biofilms on the surface of gastric mucosa, suggesting one possible explanation for eradication therapy failure. Finally, based on the results of *in vitro* analyses, *H. pylori* biofilm formation can decrease susceptibility to antibiotics and *H. pylori* antibiotic resistance mutations are more frequently generated in biofilms than in planktonic cells. These observations indicated that *H. pylori* biofilm formation may play an important role in preventing and controlling *H. pylori* infections. Therefore, investigation of *H. pylori* biofilm formation could be effective in elucidating the detailed mechanisms of infection and colonization by this microorganism.

## 1. Introduction

*Helicobacter pylori* is a spiral, microaerophilic, noninvasive, gram-negative bacterium that colonizes the human gastrointestinal tract, primarily the stomach [1].* H. pylori* is one of the most common causes of human infection, especially in developing countries, where the incidence can be up to 90% of the population [2].* H. pylori *infection often persists throughout life. This organism has been identified as an etiological agent of chronic active gastritis, peptic ulcer disease [3, 4], gastric adenocarcinoma [5], and mucosa-associated lymphoid tissue (MALT) lymphoma [6]. In addition, a working group of the World Health Organization International Agency for Research on Cancer concluded in 1994 that* H. pylori* is a group I definite carcinogen in humans [7]. Even though most individuals infected with* H. pylori* are asymptomatic, infected individuals form a high-risk population for the above-mentioned diseases. A number of factors such as the vacuolating cytotoxin, the* cagA* and* cag* pathogenicity island (*cag*PAI), motility, adhesins, and the urease enzyme are known to be involved in the virulence of this organism [8].* H. pylori* exists in two morphological forms [9]. One is a spiral form and the other is a nonculturable but viable coccoid form. The spiral form is the most common form involved in colonization of the human stomach. It has been reported that, for survival under unsuitable conditions, this microorganism has the ability to convert its spiral form to the coccoid form [9–13].

Recently, some studies have alluded to the ability of* H. pylori *to form biofilms* in vitro* [14–16]. In addition,* H. pylori *can form biofilms on the human gastric mucosa [17–19]. Moreover,* H. pylori* could be embedded in drinking water biofilms on the surfaces of water distribution systems in developed and developing countries [20]. Therefore, a more thorough understanding of* H. pylori* biofilm should provide useful information for the characterization of this microorganism. In this review, several scientific observations including our research data on* H. pylori *biofilm formation will be described. In addition, a novel eradication strategy for* H. pylori* biofilm will be suggested.

## 2. Bacterial Biofilm Formation

Most bacteria live under severe nutrient-limited conditions. To protect themselves from hostile environmental influences, bacteria often form surface attached communities described as “bacterial biofilms.” Biofilms are ubiquitous in natural, industrial, and clinical environments and have been shown to play a critical role in many chronic infections [21]. Biofilms are usually composed of multiple bacterial species. For example, dental biofilms (i.e., dental plaque) contain more than 500 different bacterial species [22]. Biofilms consist of viable microbial cells along with dead cells and a wide range of self-generated extracellular polymeric substances (EPS) including polysaccharides, nucleic acids (extracellular DNA from bacteria), and proteins [23]. The EPS matrix can constitute up to 90% of the biofilm biomass. The initial attachment is driven by hydrophobic or electrostatic interactions as well as specific bacterial surface molecules. The next step is multiplication of the bacteria and formation of microcolonies with EPS surrounding the microcolonies. In the third step (maturation step), the biofilm forms thick and mushroom-like or tower-like structures with increasing numbers of bacteria. Subsequently, the enlarged biofilm shows focal dissolution and liberates planktonic bacterial cells which can spread to other locations.

Biofilm bacteria exhibit distinct properties which differ from those of planktonic cells [24, 25]. One of these is an increased resistance to antimicrobial agents [26]. The susceptibility of biofilm cells to antimicrobial agents has been shown to differ from that of planktonic cultures [24] and this is a major contributor to the etiology of infectious diseases. In addition, another distinctive property is that biofilm cells exhibited different pattern of gene expression including the expression of virulence factor genes [27]. This property can involve a cell-to-cell communication system called quorum sensing (QS) [28]. The signaling molecules are known as autoinducers (AIs). When these molecules reach a critical threshold concentration, a signal transduction cascade is triggered. Signaling by AIs in the QS system forms the basis for alterations in various gene expressions including virulence factors, secretion system, motility, sporulation, and biofilm formation [29]. Three QS molecules were well characterized (oligopeptides, AI-1, and AI-2). Oligopeptides are produced by gram-positive bacteria and their action is species-specific. Many gram-negative bacteria utilize N-acyl-L-homoserine lactone (N-AHL) molecules as AI-1 signaling molecules [30], and these activities are also species-specific. A wide range of gram-positive and gram-negative bacterial species utilize AI-2 signaling molecules which are furanosyl borate diesters, and the enzyme responsible for their synthesis is encoded by the* luxS* gene [31, 32]. These AI systems play important roles in bacterial biofilm formation.

## 3. The Properties of* H. pylori *Biofilms

**Figure 1:**
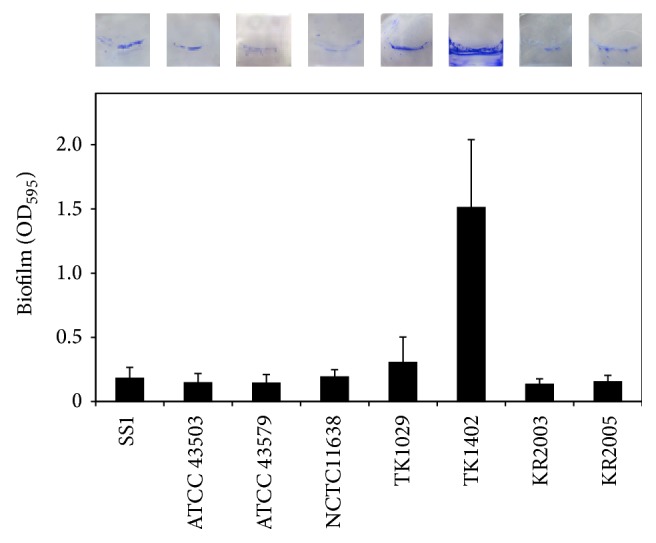
Biofilm formation by* H. pylori *strains. The graph shows quantification of biofilms formed after 3 days following culture in* Brucella* broth supplemented with 7% FCS. The upper photographs show typical biofilms on glass coverslips.

**Figure 2:**
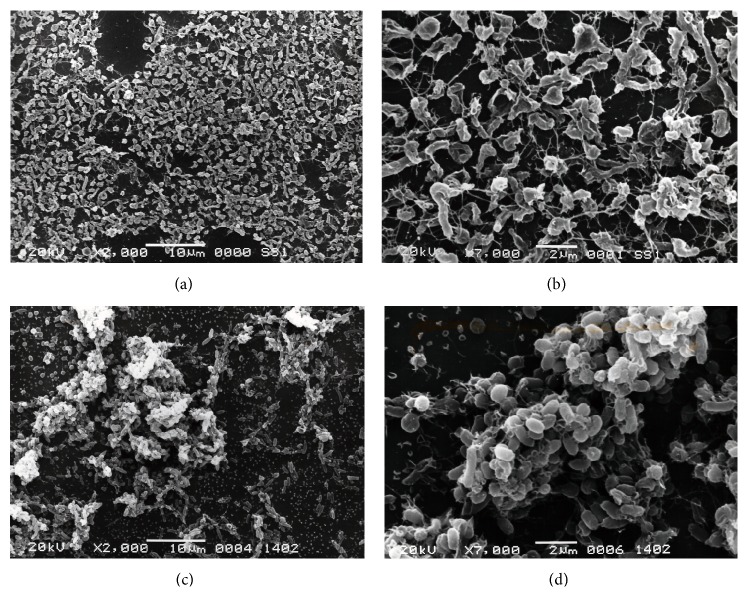
SEM images of* H. pylori* strains SS1 ((a) and (b)) and TK1402 ((c) and (d)) biofilms. The 3-day biofilm of each strain on cover glass was investigated using SEM. Photographs were taken at low (×2000; (a) and (c)) or high (×7000; (b) and (d)) magnification. Scale bar (2 *μ*m) is shown at the bottom of each electron micrograph.

In an initial investigation on biofilm formation by* H. pylori* two studies characterized biofilm formation by this organism [14, 15]. As the first demonstration of the* in vitro* ability to form biofilms by* H. pylori*, Stark et al. reported that a water insoluble polysaccharide-containing biofilm has been observed at the air-liquid interface when* H. pylori* strain NCTC 11637 was continuously grown in a glass fermenter [14]. Subsequently, Cole et al. reported that all of the* H. pylori* strains used in their study, including clinical isolates, laboratory strains, and a mouse-adapted strain, were able to form biofilms on glass surfaces [15]. They also reported that* H. pylori* could form a biofilm only at the air-liquid interface, which is most likely indicative of its microaerobicity. However, at present, biofilm formation by* H. pylori *has not been extensively characterized. Therefore, we analyzed the ability of* H. pylori* strains to form biofilms and characterized the underlying mechanisms involved. Initially, we established a feasible and stable model for biofilm formation by this microorganism. Briefly, sterilized glass coverslips were placed into 12-well microtiter plates. Each well was filled with 2 mL of* Brucella* broth supplemented with 7% fetal calf serum (FCS) to allow adherence of* H. pylori* at the air-liquid interface. The formation of biofilms was initiated by inoculating approximately 5 × 10^5^ cells into each well. The cultures were incubated under microaerobic conditions at 37°C for 3 to 5 days with shaking. Using this model, the biofilm forming ability of eight* H. pylori* strains including standard SS1, ATCC 43579, ATCC 43579, and NCTC11638 strains and clinical isolates from Japanese patients was analyzed. Under these conditions, all of the strains formed biofilms at the liquid-gas interface of the cultures. Specifically, strain TK1402, which was isolated from a Japanese patient with duodenal and gastric ulcers, showed significantly higher levels of biofilm formation relative to the other strains (Figure 1) [33]. The strong biofilm forming ability of TK1402 was reflected in the relative thickness of the biofilms. To clarify the architectural characteristics of* H. pylori* biofilms, we compared TK1402 and SS1 biofilms by scanning electron microscopy (SEM) (Figure 2) [34]. In the SS1 biofilms, the bacteria attached to glass surfaces in thin layers, and the biofilms consisted mainly of bleb-like or amorphous structures (Figures 2(a) and 2(b)). On the other hand, the TK1402 biofilms were composed primarily of cells with bacillary morphology which were clearly outlined (Figures 2(c) and 2(d)). We also analyzed the biofilm cells of the other strains using SEM. However, the majority of these biofilm cells consisted of autolysed cells, suggesting that the strong biofilm forming ability of TK402 may have resulted from an active metabolic state for a relatively long time without exhibiting morphological changes or autolysis. In addition, the biofilms of TK1402 strain showed the presence of many outer membrane vesicles (OMVs) on the glass surfaces as well as on the bacterial cell surfaces. These structures were not detected in the biofilms of the other strains. OMVs were more closely observed in the thin-sectioned biofilms using transmission electron microscopy (TEM) and the OMVs were located at the substratum-bacterium interface and in the extracellular spaces. In addition, biofilm formation by strain TK1402 was strongly correlated with the production of OMV. These results suggested that the OMV produced by strain TK1402 may serve as an EPS matrix for these biofilms. OMV production is a physiologically normal function of gram-negative bacteria [35, 36]. In* Pseudomonas aeruginosa*, OMVs have multifunctional biological roles including microbial interaction and host infection as well as maintenance of the structure of biofilm [37, 38]. In* Porphyromonas gingivalis*, OMVs promote attachment, aggregation, and biofilm formation and the functions of OMVs in biofilms have been discussed [39, 40]. Similar to most gram-negative bacteria,* H. pylori* released OMV into the extracellular space [41, 42]. Major protein and phospholipid components associated with the OMVs were identified [43]. We analyzed the protein profile of the OMV produced by strain TK1402 to determine which components of the OMV contribute to biofilm formation in* H. pylori.* The results indicated that a specific approximately 22 kDa protein might be involved in the biofilm forming ability of this strain [44]. Additional research is now in progress to determine what factors are directly involved in biofilm formation by strain TK1402.

Concerning the* H. pylori* biofilm matrix, Grande et al. demonstrated that extracellular DNA is a component of EPS structures and is important in stabilizing biofilm structures [45]. Yang et al. indicated that mannose-related proteoglycans (proteomannans) are one component of the EPS structures and proteomannans are also involved in the process of* H. pylori* biofilm formation [46]. They also reported that the neutrophil-activating protein A (NapA) is upregulated in biofilm cells compared to planktonic cells, and biofilm formation with a* napA* deficient mutant exhibited a different phenotypic biofilm. Recently, Grande et al. demonstrated that biofilms developed by multiple* H. pylori* strains are more complex than those associated with single strains and such conditions might promote genetic exchange favoring the generation of more virulent strains [47].

## 4. Quorum Sensing in* H. pylori*

The* luxS* gene is the only known quorum-sensing gene present in the sequenced* H. pylori* genome. Several reports indicated that* H. pylori* produces extracellular signaling molecules related to AI-2, and production of AI-2 is dependent on* luxS* function [48–50]. These reports have indicated that the production of AI-2 by* luxS* is growth-phase dependent, with maximal production occurring in the mid-exponential phase of growth. Several reports indicated that LuxS has an alternative role in regulation of motility by modulating flagellar transcription and flagellar biosynthesis [51, 52]. Our previous study also demonstrated that strain TK1402* luxS* deficient mutant exhibited significantly lower motility than that of parental strain [53]. In addition, the* luxS* mutant exhibited a reduced infection rate relative to the wild-type parent strain TK1402 in a Mongolian gerbil model. Cole et al. reported the relations of* luxS* quorum sensing and biofilm formation in* H. pylori* [15]. They demonstrated that the* luxS* mutants of clinically isolated strains, SD3 and SD4, were approximately twofold more better at forming a biofilm than the parental strains. On the other hand, Doherty et al. indicated that LuxS fulfills primarily a metabolic role in the activated methyl cycle, which generates the* S*-adenosylmethionine required by methyltransferases and recycles the product via methionine as well as cell-to-cell signaling [54]. Further investigations are expected to elucidate the function of LuxS.

## 5. *H. pylori* Biofilm Formation in the Environment

The principal mode of transmission proposed for* H. pylori* is person to person contact via the faecal-oral, oral-oral, or gastro-oral routes [55–58]. However, especially in developing countries, the patterns of* H. pylori* transmission suggest a universal source for exposure rather than person to person transmission [59]. Thus, the drinking water supply was highlighted as an important source of* H. pylori* infection and, indeed,* H. pylori* was only detected with special procedures in water distribution systems [60, 61]. In addition, the role of water sources and associated biofilms acting as environmental transmitters of* H. pylori* has been suggested by the detection of* H. pylori* DNA by molecular methods, such as PCR, in sewage, well water, pond and river water, river water, and shallow ground water in developed countries as well as in developing countries [61–66]. These data suggested that* H. pylori* exists in water distribution systems and that the organism may survive in biofilms in these systems. However, in fact, it does not appear that* H. pylori* forms biofilms at locations which are relatively stressful conditions such as less than optimal temperatures and nutrient limitation. In oligotrophic water systems, the bacterial genera* Pedomicrobium*,* Hyphomicrobium*,* Gallionella*, and* Caulobacter* were regularly found [67]. It is likely that these bacteria form biofilms in drinking water distribution systems and are then contaminated with* H. pylori* from sewage, well water, pond and river water, river water, and shallow ground water and are embedded in such bacterial biofilm structures. Indeed,* H. pylori* has never been cultured from drinking water distribution systems using standard cultivation techniques [68, 69]. These reports indicated that it is impossible to distinguish between alive and dead cells of* H. pylori* in such systems. Recently, it was reported with several new methods such as in situ fluorescent hybridization (FISH) [20, 70] to detect viable* H. pylori* in various water sources. Continuous critical investigation is necessary as it remains unclear to what extent there is a health risk from this source.

## 6. *H. pylori* Biofilm Formation on Human Gastric Mucosa

The first photographic documentation of the existence of* H. pylori* biofilms on human gastric mucosa was reported by Carron et al. using endoscopically directed biopsies and scanning electron microscopy [17]. Mature biofilms were present and attached to the cell surface of* H. pylori*-positive specimens. Their group subsequently reported that, among patients with peptic ulcer disease who were tested urease positive for* H. pylori*, the average rate of total cell surfaces covered by biofilms was 97.3%, as opposed to 1.64% for urease-negative patients [18]. Cellini et al. reported that a prevalent S-shape* H. pylori* morphotype which coexisted with coccid aggregated bacteria embedded in an abundant matrix was demonstrated by SEM analysis with biopsies from patients harboring culturable bacteria [19]. On the other hand, samples from patients shown as* H. pylori*-positive only through the molecular methods showed clustered coccid bacteria arranged in a microbial biofilm. Cammarota et al. reported that, among the patients who had a history of at least four* H. pylori* eradication failures, SEM analysis of gastric biopsies showed that* H. pylori* formed biofilms on the gastric mucosa in all of the patients and that the biofilm disappeared in all of them when the microorganism was eradicated [71].

## 7. Effects of* H. pylori* Biofilms on Susceptibility to Antimicrobial Agents

Eradication of* H. pylori* is important not only for the treatment of gastric/duodenal ulcer, but also for the treatment and prevention of* H. pylori*-associated diseases such as gastric cancer, as well as for inhibiting the spread of this microorganism. For the eradication of* H. pylori*, a combination therapy using an antiacid agent (proton pump inhibitor (PPI) or H_2_ blocker) and two anti-*H. pylori* agents (amoxicillin and either clarithromycin (CAM) or metronidazole) has been recommended [72–74]. Fluoroquinolones have also been selected as anti-*H. pylori* agents. In Japan, a combination of a proton pump inhibitor, amoxicillin, and CAM is commonly used in first-line eradication therapy [72]. However, CAM resistance is an increasing problem for the first-line therapy of* H. pylori* infection, since the major cause of eradication failure is thought to be the existence of CAM resistant* H. pylori* [72, 74–77]. CAM resistant* H. pylori* are extremely common and the frequency of CAM resistant clinical isolates ranges from approximately 10 to 30% [74, 78]. Point mutations in the domain V loop of the 23S rRNA gene (commonly an adenine-to-guanine transition at position 2142 or 2143) have been reported as the basis for resistance [72, 74–79].

In other bacterial biofilms, biofilm grown cells express properties distinct from planktonic cells, one of which is an increased resistance to antimicrobial agents [26, 80–83]. Based on these reports, the biofilm cells can become 10–1000 times more resistant to the effects of antimicrobial agents. Multiple mechanisms of biofilm resistance to antimicrobial compounds were suggested: (i) failure of the antimicrobial compounds to penetrate the biofilm, (ii) slow growth of the biofilm cells owing to nutrient limitation, and (iii) activation of the general stress response [26, 84–88]. However, the effect of* H. pylori* biofilm formation on antibiotics susceptibility is not well documented. Thus, we investigated the effects of CAM on* H. pylori* biofilms [89]. Biofilm formation in* H. pylori* increased the resistance to CAM at minimum inhibitory concentration (MIC) levels by up to 4-fold in 2-day biofilms (intermediated biofilms) and to 16-fold in 3-day biofilms (mature biofilms) as well as minimum bactericidal concentration (MBC) levels by up to 4-fold compared to planktonic cells. Participation of the efflux pumps of the resistance-nodulation-cell division (RND) family was involved in the development of CAM resistance in* H. pylori* biofilm and failure of CAM penetration into the biofilm interior due to the presence of the extracellular matrix was also demonstrated. In addition, we demonstrated that* H. pylori* biofilm formation can affect the generation of CAM resistance mutations (Table 1). CAM resistant cells were detected more frequently in biofilms after treatment with CAM. Our results indicated that the relatively high concentration, especially one-quarter of MBC (0.25 *μ*g/mL, which are concentrations equivalent to 16x MIC), of CAM may facilitate the generation of CAM resistance mutations in* H. pylori* biofilms.

## 8. Therapy for Preventing* H. pylori* Biofilm Infection

Antibiotic resistance in* H. pylori* can therefore be acquired by the selection of spontaneous mutation events that occur due to the magnitude and duration of antibiotic use on the human gastric mucosa. Nakamura et al. reported that CAM concentrations in gastric juices, mucosa, or serum after administration of 500 mg of the drug for 7 days were 550.6, 64.6, and 2.5 *μ*g/mL at 2 hours after administration and 43.4, 36.2, and 2.2 *μ*g/mL at 6 hours, respectively [90]. These concentrations might be sufficient to reduce the levels of* H. pylori in vivo* so that this microorganism formed biofilms. However, to reach such high concentrations of CAM on the gastric mucosa for extended periods, the drug needs to be taken with sufficient dosage. In addition, in cases with inadequate compliance with eradication therapy, the concentration of CAM does not reach high levels in the gastric mucosa. Further, macrolides including CAM are frequently used in the treatment of various infectious diseases in pediatric, respiratory, and otorhinolaryngology settings. In these cases, biofilm formation by* H. pylori* may contribute to the acquisition of CAM resistance.

Novel approaches to prevent biofilm formation and to treat infections by biofilm-forming bacteria are currently under development [91, 92]. Recently, a clinical trial for effective strategies targeting* H. pylori* biofilm infections through the use of molecules such as* N*-acetylcysteine (NAC) was reported [71, 93]. NAC is a mucolytic and a thiol-containing antioxidant agent and is considered a nonantibiotic drug that has antibacterial properties. In 1977, Parry and Neu found that NAC had the ability to inhibit the growth of both gram-positive and gram-negative bacteria, including* Staphylococcus aureus*,* P. aeruginosa*,* Klebsiella pneumoniae,* and* Enterobacter cloacae *[94]. The antibacterial effect of NAC may be due to competitively inhibiting amino acid (cysteine) utilization or by virtue of possessing a sulfhydryl group it may react with bacterial cell proteins. Moreover, previous studies demonstrated decreased biofilm formation by a variety of bacteria in the presence of NAC [95–98], leading to an inhibition of bacterial adherence, a reduction in the production of the extracellular polysaccharide matrix promoting the disruption of mature biofilms, and a reduction in sessile cell viability [95–98]. Relative to* H. pylori* biofilms, NAC is effective in both inhibiting* H. pylori* biofilm formation and disrupting developed biofilms* in vitro* [71]. In addition, NAC treatment preceding the initiation of antibiotic eradication therapy is able to provide eradication of resistant* H. pylori* infections. Large scale studies regarding the effectiveness of NAC* in vivo* for reducing* H. pylori* biofilms are still required.

## 9. Conclusions

Pathogenic bacteria including* H. pylori* within biofilms can escape from both host immune responses and the effects of antimicrobial agents. Consequently, chronic infections by biofilm forming bacteria become troublesome and difficult to treat. Some of the previous studies have shown that* H. pylori* forms biofilm on human gastric mucosa. Nevertheless, assessment of* H. pylori* strain susceptibility to antibiotics* in vitro* has traditionally been evaluated using planktonic cells, so that MICs are not reliable predictors of the antibiotic effects in the human stomach. The assessment of the ability to form biofilms in* H. pylori* could play an important role in preventing and controlling the generation of antibiotic resistance. It is expected that enhancing our knowledge of* H. pylori* biofilm formation will lead to new treatment therapies for preventing* H. pylori* infections. However, it is recognized that our understanding of* H. pylori* biofilm formation is still in its infancy. Further studies of the mechanism of* H. pylori* biofilm formation need to be performed. In addition, investigation into novel* H. pylori* eradication strategies for the human gastric mucosa using biofilm-dissolving compounds, quorum sensing inhibitors, or conventional antibiotics may provide advantages in resolving* H. pylori* infections.

## Figures and Tables

**Table 1:** Generation of CAM resistance mutations in biofilm and planktonic cells. The 2-day and 3-day biofilms and planktonic cells were exposed to the indicated concentrations of CAM (biofilms were exposed to one-eighth, one-quarter, or one-half of the MBC of CAM at concentrations of 0.125, 0.25, and 0.5 *μ*g/mL, concentrations which are equivalent to 8x, 16x, and 32x MIC and planktonic cultures were also exposed to one-quarter or one-half of the MBC of CAM at concentrations of 0.063 and 0.125 *μ*g/mL, concentrations which are equivalent to 4x and 8x MIC) for 24 h under microaerobic conditions at 37°C with shaking. After incubation, cells were recovered in fresh *Brucella* supplemented with 7% FCS agar, and the generation of CAM resistant mutants was assessed in media supplemented with 1.0 *μ*g/mL CAM. When no CAM resistant cells were detected, exposure to CAM was repeated up to 5 times. The table indicates the accumulation ratio of the generated CAM resistance in biofilms (number of samples was 12 or 13) or in planktonic cultures (number of samples was 12).

Samples	Passage time				
CAM concentrations	1st	2nd	3rd	4th	5th
2-day biofilm					
CAM 0.5 *μ*g/mL	0/12 (0%)	0/12 (0%)	1/12 (8%)	2/12 (17%)	4/12 (33%)
CAM 0.25 *μ*g/mL	1/12 (8%)	4/12 (33%)	6/12 (50%)	8/12 (67%)	9/12 (75%)
CAM 0.125 *μ*g/mL	0/12 (0%)	1/12 (8%)	2/12 (17%)	3/12 (25%)	4/12 (33%)
2-day planktonic					
CAM 0.125 *μ*g/mL	0/12 (0%)	0/12 (0%)	1/12 (8%)	4/12 (33%)	4/12 (33%)
CAM 0.063 *μ*g/mL	0/12 (0%)	0/12 (0%)	3/12 (25%)	3/12 (25%)	3/12 (25%)
3-day biofilm					
CAM 0.5 *μ*g/mL	1/12 (8%)	3/12 (25%)	4/12 (33%)	6/12 (50%)	6/12 (50%)
CAM 0.25 *μ*g/mL	1/13 (8%)	5/13 (38%)	11/13 (85%)	11/13 (85%)	11/13 (85%)
CAM 0.125 *μ*g/mL	1/13 (8%)	2/13 (15)	3/13 (23%)	5/13 (38%)	6/13 (46%)
3-day planktonic					
CAM 0.125 *μ*g/mL	0/12 (0%)	1/12 (8%)	1/12 (8%)	1/12 (8%)	3/12 (25%)
CAM 0.063 *μ*g/mL	1/12 (8%)	1/12 (8%)	1/12 (8%)	1/12 (8%)	3/12 (25%)

## References

[1] Marshall B. J., Warren J. R. (1984). Unidentified curved bacilli in the stomach of patients with gastritis and peptic ulceration. *The Lancet*.

[2] Dunn B. E., Cohen H., Blaser M. J. (1997). Helicobacter pylori. *Clinical Microbiology Reviews*.

[3] Blaser M. J. (1992). *Helicobacter pylori*: its role in disease. *Clinical Infectious Diseases*.

[4] Graham D. Y. (1989). *Campylobacter pylori* and peptic ulcer disease. *Gastroenterology*.

[5] Parsonnet J., Friedman G. D., Vandersteen D. P. (1991). *Helicobacter pylori* infection and the risk of gastric carcinoma. *The New England Journal of Medicine*.

[6] Wotherspoon A. C., Doglioni C., Diss T. C. (1993). Regression of primary low-grade-B-cell gastric lymphoma of mucosa-associated lymphoid tissue type after eradication of *Helicobacter pylori*. *The Lancet*.

[7] International Agency for Research on Cancer (1994). Schistosomes, liver flukes, and *Helicobacter pylori*. *Monographs on the Evaluation of Carcinogenic Risks to Humans*.

[8] Bijlsma J. J. E., Vandenbroucke-Grauls C. M., Phadnis S. H., Kusters J. G. (1999). Identification of virulence genes of *Helicobacter pylori* by random insertion mutagenesis. *Infection and Immunity*.

[9] Bode G., Mauch F., Malfertheiner P. (1993). The coccoid forms of *Helicobacter pylori*. Criteria for their viability. *Epidemiology and Infection*.

[10] Cellini L., Allocati N., Di Campli E., Dainelli B. (1994). *Helicobacter pylori*: a fickle germ. *Microbiology and Immunology*.

[11] Mizoguchi H., Fujioka T., Nasu M. (1999). Evidence for viability of coccoid forms of *Helicobacter pylori*. *Journal of Gastroenterology*.

[12] Reynolds D. J., Penn C. W. (1994). Characteristics of *Helicobacter pylori* growth in a defined medium and determination of its amino acid requirements. *Microbiology*.

[13] Shahamat M., Mai U., Paszko-Kolva C., Kessel M., Colwell R. R. (1993). Use of autoradiography to assess viability of *Helicobacter pylori* in water. *Applied and Environmental Microbiology*.

[14] Stark R. M., Gerwig G. J., Pitman R. S. (1999). Biofilm formation by *Helicobacter pylori*. *Letters in Applied Microbiology*.

[15] Cole S. P., Harwood J., Lee R., She R., Guiney D. G. (2004). Characterization of monospecies biofilm formation by *Helicobacter pylori*. *Journal of Bacteriology*.

[16] Cellini L., Grande R., di Campli E. (2008). Characterization of an *Helicobacter pylori* environmental strain. *Journal of Applied Microbiology*.

[17] Carron M. A., Tran V. R., Sugawa C., Coticchia J. M. (2006). Identification of *Helicobacter pylori* biofilms in human gastric mucosa. *Journal of Gastrointestinal Surgery*.

[18] Coticchia J. M., Sugawa C., Tran V. R., Gurrola J., Kowalski E., Carron M. A. (2006). Presence and density of *Helicobacter pylori* biofilms in human gastric mucosa in patients with peptic ulcer disease. *Journal of Gastrointestinal Surgery*.

[19] Cellini L., Grande R., Campli E. D. (2008). Dynamic colonization of *Helicobacter pylori* in human gastric mucosa. *Scandinavian Journal of Gastroenterology*.

[20] García A., Salas-Jara M. J., Herrera C., González C. (2014). Biofilm and *Helicobacter pylori*: from environment to human host. *World Journal of Gastroenterology*.

[21] Parsek M. R., Singh P. K. (2003). Bacterial biofilms: an emerging link to disease pathogenesis. *Annual Review of Microbiology*.

[22] Whittaker C. J., Klier C. M., Kolenbrander P. E. (1996). Mechanisms of adhesion by oral bacteria. *Annual Review of Microbiology*.

[23] Whitchurch C. B., Tolker-Nielsen T., Ragas P. C., Mattick J. S. (2002). Extracellular DNA required for bacterial biofilm formation. *Science*.

[24] Costerton J. W., Stewart P. S., Greenberg E. P. (1999). Bacterial biofilms: a common cause of persistent infections. *Science*.

[25] O'Toole G., Kaplan H. B., Kolter R. (2000). Biofilm formation as microbial development. *Annual Review of Microbiology*.

[26] Mah T.-F. C., O'Toole G. A. (2001). Mechanisms of biofilm resistance to antimicrobial agents. *Trends in Microbiology*.

[27] Davies D. G., Chakrabarty A. M., Geesey G. G. (1993). Exopolysaccharide production in biofilms: Substratum activation of alginate gene expression by *Pseudomonas aeruginosa*. *Applied and Environmental Microbiology*.

[28] Sperandio V., Torres A. G., Jarvis B., Nataro J. P., Kaper J. B. (2003). Bacteria-host communication: the language of hormones. *Proceedings of the National Academy of Sciences of the United States of America*.

[29] Fuqua W. C., Winans S. C., Greenberg E. P. (1994). Quorum sensing in bacteria: the LuxR-LuxI family of cell density—responsive transcriptional regulators. *Journal of Bacteriology*.

[30] Fuqua C., Parsek M. R., Greenberg E. P. (2001). Regulation of gene expression by cell-to-cell communication: acyl-homoserine lactone quorum sensing. *Annual Review of Genetics*.

[31] Surette M. G., Bassler B. L. (1999). Regulation of autoinducer production in *Salmonella typhimurium*. *Molecular Microbiology*.

[32] Chen X., Schauder S., Potier N. (2002). Structural identification of a bacterial quorum-sensing signal containing boron. *Nature*.

[33] Yonezawa H., Osaki T., Kurata S., Zaman C., Hanawa T., Kamiya S. (2010). Assessment of *in vitro* biofilm formation by *Helicobacter pylori*. *Journal of Gastroenterology and Hepatology*.

[34] Yonezawa H., Osaki T., Kurata S. (2009). Outer membrane vesicles of *Helicobacter pylori* TK1402 are involved in biofilm formation. *BMC Microbiology*.

[35] Lee E.-Y., Choi D.-S., Kim K.-P., Gho Y. S. (2008). Proteomics in Gram-negative bacterial outer membrane vesicles. *Mass Spectrometry Reviews*.

[36] Beveridge T. J. (1999). Structures of gram-negative cell walls and their derived membrane vesicles. *Journal of Bacteriology*.

[37] Schooling S. R., Beveridge T. J. (2006). Membrane vesicles: an overlooked component of the matrices of biofilms. *Journal of Bacteriology*.

[38] Tashiro Y., Uchiyama H., Nomura N. (2012). Multifunctional membrane vesicles in *Pseudomonas aeruginosa*. *Environmental Microbiology*.

[39] Inagaki S., Onishi S., Kuramitsu H. K., Sharma A. (2006). *Porphyromonas gingivalis* vesicles enhance attachment, and the leucine-rich repeat BspA protein is required for invasion of epithelial cells by ‘*Tannerella forsythia*’. *Infection and Immunity*.

[40] Yamaguchi M., Sato K., Yukitake H., Noiri Y., Ebisu S., Nakayama K. (2010). A *Porphyromonas gingivalis* mutant defective in a putative glycosyltransferase exhibits defective biosynthesis of the polysaccharide portions of lipopolysaccharide, decreased gingipain activities, strong autoaggregation, and increased biofilm formation. *Infection and Immunity*.

[41] Fiocca R., Necchi V., Sommi P. (1999). Release of* Helicobacter pylori* vacuolating cytotoxin by both a specific secretion pathway and budding of outer membrane vesicles. Uptake of released toxin and vesicles by gastric epithelium. *The Journal of Pathology*.

[42] Keenan J. I., Allardyce R. A., Bagshaw P. F. (1997). Dual silver staining to characterise *Helicobacter* spp. outer membrane components. *Journal of Immunological Methods*.

[43] Olofsson A., Vallström A., Petzold K. (2010). Biochemical and functional characterization of *Helicobacter pylori* vesicles. *Molecular Microbiology*.

[44] Yonezawa H., Osaki T., Woo T. (2011). Analysis of outer membrane vesicle protein involved in biofilm formation of *Helicobacter pylori*. *Anaerobe*.

[45] Grande R., di Giulio M., Bessa L. J. (2011). Extracellular DNA in *Helicobacter pylori* biofilm: a backstairs rumour. *Journal of Applied Microbiology*.

[46] Yang F.-L., Hassanbhai A. M., Chen H.-Y. (2011). Proteomannans in Biofilm of *Helicobacter pylori* ATCC 43504. *Helicobacter*.

[47] Grande R., Di Campli E., Di Bartolomeo S. (2012). *Helicobacter pylori* biofilm: a protective environment for bacterial recombination. *Journal of Applied Microbiology*.

[48] Forsyth M. H., Cover T. L. (2000). Intercellular communication in *Helicobacter pylori*: *luxS* is essential for the production of an extracellular signaling molecule. *Infection and Immunity*.

[49] Joyce E. A., Bassler B. L., Wright A. (2000). Evidence for a signaling system in *Helicobacter pylori*: detection of a *luxS*-encoded autoinducer. *Journal of Bacteriology*.

[50] Lee W. K., Ogura K., Loh J. T., Cover T. L., Berg D. E. (2006). Quantitative effect of *luxS* gene inactivation on the fitness of *Helicobacter pylori*. *Applied and Environmental Microbiology*.

[51] Rader B. A., Wreden C., Hicks K. G., Sweeney E. G., Ottemann K. M., Guillemin K. (2011). *Helicobacter pylori* perceives the quorum-sensing molecule AI-2 as a chemorepellent via the chemoreceptor TlpB. *Microbiology*.

[52] Shen F., Hobley L., Doherty N. (2010). In *Helicobacter pylori* auto-inducer-2, but not LuxS/MccAB catalysed reverse transsulphuration, regulates motility through modulation of flagellar gene transcription. *BMC Microbiology*.

[53] Osaki T., Hanawa T., Manzoku T. (2006). Mutation of *luxS* affects motility and infectivity of *Helicobacter pylori* in gastric mucosa of a Mongolian gerbil model. *Journal of Medical Microbiology*.

[54] Doherty N. C., Shen F., Halliday N. M. (2010). In *Helicobacter pylori*, LuxS is a key enzyme in cysteine provision through a reverse transsulfuration pathway. *Journal of Bacteriology*.

[55] Thomas J. E., Gibson G. R., Darboe M. K., Dale A., Weaver L. T. (1992). Isolation of *Helicobacter pylori* from human faeces. *The Lancet*.

[56] Madinier I. M., Fosse T. M., Monteil R. A. (1997). Oral Carriage of *Helicobacter pylori*: a review. *Journal of Periodontology*.

[57] Parsonnet J., Shmuely H., Haggerty T. (1999). Fecal and oral shedding of *Helicobacter pylori* from healthy infected adults. *The Journal of the American Medical Association*.

[58] Osaki T., Okuda M., Ueda J. (2013). Multilocus sequence typing of DNA from faecal specimens for the analysis of intra-familial transmission of *Helicobacter pylori*. *Journal of Medical Microbiology*.

[59] Akcan Y., Ersan S., Alper M., Bjcjk Z., Aytug N. (2000). The transmission of *Helicobacter pylori* via exposure to common sources outweighs the person-to-person contact among spouses in developing countries. *The American Journal of Gastroenterology*.

[60] Klein P. D., Graham D. Y., Gaillour A. (1991). Water source as risk factor for *Helicobacter pylori* infection in Peruvian children. *The Lancet*.

[61] Watson C. L., Owen R. J., Said B. (2004). Detection of *Helicobacter pylori* by PCR but not culture in water and biofilm samples from drinking water distribution systems in England. *Journal of Applied Microbiology*.

[62] Hegarty J. P., Dowd M. T., Baker K. H. (1999). Occurrence of *Helicobacter pylori* in surface water in the United States. *Journal of Applied Microbiology*.

[63] Horiuchi T., Ohkusa T., Watanabe M., Kobayashi D., Miwa H., Eishi Y. (2001). *Helicobacter pylori* DNA in drinking water in Japan. *Microbiology and Immunology*.

[64] Imanishi Y., Ogata T., Matsuzuka A. (2003). Possibility for the presence of *Helicobacter pylori* in drinking well water. *Kansenshogaku Zasshi*.

[65] Lu Y., Redlinger T. E., Avitia R., Galindo A., Goodman K. (2002). Isolation and genotyping of *Helicobacter pylori* from untreated municipal wastewater. *Applied and Environmental Microbiology*.

[66] Moreno Y., Botella S., Alonso J. L., Ferrús M. A., Hernández M., Hernández J. (2003). Specific detection of *Arcobacter* and *Campylobacter* strains in water and sewage by PCR and fluorescent in situ hybridization. *Applied and Environmental Microbiology*.

[67] Szewzyk U., Szewzyk R., Manz W., Schleifer K. H. (2000). Microbiogical safety of drinking water. *Annual Review of Microbiology*.

[68] Azevedo N. F., Guimarães N., Figueiredo C., Keevil C. W., Vieira M. J. (2007). A new model for the transmission of *Helicobacter pylori*: role of environmental reservoirs as gene pools to increase strain diversity. *Critical Reviews in Microbiology*.

[69] Gião M. S., Azevedo N. F., Wilks S. A., Vieira M. J., Keevil C. W. (2008). Persistence of *Helicobacter pylori* in heterotrophic drinking-water biofilms. *Applied and Environmental Microbiology*.

[70] Percival S. L., Suleman L. (2014). Biofilms and *Helicobacter pylori*: dissemination and persistence within the environment and host. *World Journal of Gastrointestinal Pathophysiology*.

[71] Cammarota G., Branca G., Ardito F. (2010). Biofilm demolition and antibiotic treatment to eradicate resistant *Helicobacter pylori*: a clinical trial. *Clinical Gastroenterology and Hepatology*.

[72] Asaka M., Kato M., Takahashi S.-I. (2010). Guidelines for the management of *Helicobacter pylori* infection in Japan: 2009 revised edition. *Helicobacter*.

[73] Egan B. J., Marzio L., O'Connor H., O'Morain C. (2008). Treatment of *Helicobacter pylori* infection. *Helicobacter*.

[74] Malfertheiner P., Megraud F., O'Morain C. A. (2012). Management of *Helicobacter pylori* infection—the Maastricht IV/ Florence consensus report. *Gut*.

[75] Graham D. Y., de Boer W. A., Tytgat G. N. J. (1996). Choosing the best anti-*Helicobacter pylori* therapy: effect of antimicrobial resistance. *The American Journal of Gastroenterology*.

[76] Adamek R. J., Suerbaum S., Pfaffenbach B., Opferkuch W. (1998). Primary and acquired *Helicobacter priori* resistance to clarithromycin, metronidazole, and amoxicillin—influence on treatment outcome. *American Journal of Gastroenterology*.

[77] Mégraud F., Doermann H. P. (1998). Clinical relevance of resistant strains of *Helicobacter pylori*: a review of current data. *Gut*.

[78] Horiki N., Omata F., Uemura M. (2009). Annual change of primary resistance to clarithromycin among *Helicobacter pylori* isolates from 1996 through 2008 in Japan. *Helicobacter*.

[79] Versalovic J., Osato M. S., Spakovsky K. (1997). Point mutations in the 23S rRNA gene of *Helicobacter pylori* associated with different levels of clarithromycin resistance. *Journal of Antimicrobial Chemotherapy*.

[80] Prosser B. L. T., Taylor D., Dix B. A., Cleeland R. (1987). Method of evaluating effects of antibiotics on bacterial biofilm. *Antimicrobial Agents and Chemotherapy*.

[81] Nickel J. C., Ruseska I., Wright J. B., Costerton J. W. (1985). Tobramycin resistance of *Pseudomonas aeruginosa* cells growing as a biofilm on urinary catheter material. *Antimicrobial Agents and Chemotherapy*.

[82] Gristina A. G., Hobgood C. D., Webb L. X., Myrvik Q. N. (1987). Adhesive colonization of biomaterials and antibiotic resistance. *Biomaterials*.

[83] Evans R. C., Holmes C. J. (1987). Effect of vancomycin hydrochloride on *Staphylococcus epidermidis* biofilm associated with silicone elastomer. *Antimicrobial Agents and Chemotherapy*.

[84] Costerton J. W., Lewandowski Z., Caldwell D. E., Korber D. R., Lappin-Scott H. M. (1995). Microbial biofilms. *Annual Review of Microbiology*.

[85] Adams J. L., McLean R. J. C. (1999). Impact of *rpoS* deletion on *Escherichia coli* biofilms. *Applied and Environmental Microbiology*.

[86] Anderl J. N., Franklin M. J., Stewart P. S. (2000). Role of antibiotic penetration limitation in *Klebsiella pneumoniae* biofilm resistance to ampicillin and ciprofloxacin. *Antimicrobial Agents and Chemotherapy*.

[87] Desai M., Bühler T., Weller P. H., Brown M. R. W. (1998). Increasing resistance of planktonic and biofilm cultures of *Burkholderia cepacia* to ciprofloxacin and ceftazidime during exponential growth. *Journal of Antimicrobial Chemotherapy*.

[88] Dunne W. M., Mason E. O., Kaplan S. L. (1993). Diffusion of rifampin and vancomycin through a *Staphylococcus epidermidis* biofilm. *Antimicrobial Agents and Chemotherapy*.

[89] Yonezawa H., Osaki T., Hanawa T., Kurata S., Ochiai K., Kamiya S. (2013). Impact of *Helicobacter pylori* biofilm formation on clarithromycin susceptibility and generation of resistance mutations. *PLoS ONE*.

[90] Nakamura M., Spiller R. C., Barrett D. A. (2003). Gastric juice, gastric tissue and blood antibiotic concentrations following omeprazole, amoxicillin and clarithromycin triple therapy. *Helicobacter*.

[91] Römling U., Balsalobre C. (2012). Biofilm infections, their resilience to therapy and innovative treatment strategies. *Journal of Internal Medicine*.

[92] Donlan R. M., Costerton J. W. (2002). Biofilms: survival mechanisms of clinically relevant microorganisms. *Clinical Microbiology Reviews*.

[93] Cammarota G., Sanguinetti M., Gallo A., Posteraro B. (2012). Review article: biofilm formation by *Helicobacter pylori* as a target for eradication of resistant infection. *Alimentary Pharmacology and Therapeutics*.

[94] Parry M. F., Neu H. C. (1977). Effect of N-acetylcysteine on antibiotic activity and bacterial growth in vitro. *Journal of Clinical Microbiology*.

[95] Marchese A., Bozzolasco M., Gualco L., Debbia E. A., Schito G. C., Schito A. M. (2003). Effect of fosfomycin alone and in combination with N-acetylcysteine on *E. coli* biofilms. *International Journal of Antimicrobial Agents*.

[96] Pérez-Giraldo C., Rodriguez-Benito A., Morán F. J., Hurtado C., Blanco M. T., Gómez-Garcí A. C. (1997). Influence of N-acetylcysteine on the formation of biofilm by *Staphylococcus epidermidis*. *Journal of Antimicrobial Chemotherapy*.

[97] Schwandt L. Q., van Weissenbruch R., Stokroos I., van der Mei H. C., Busscher H. J., Albers F. W. J. (2004). Prevention of biofilm formation by dairy products and N-acetylcysteine on voice prostheses in an artificial throat. *Acta Oto-Laryngologica*.

[98] Olofsson A.-C., Hermansson M., Elwing H. (2003). *N*-acetyl-l-cysteine affects growth, extracellular polysaccharide production, and bacterial biofilm formation on solid surfaces. *Applied and Environmental Microbiology*.

